# Effects of 6 Months of Soy-Enriched High Protein Compared to Eucaloric Low Protein Snack Replacement on Appetite, Dietary Intake, and Body Composition in Normal-Weight Obese Women: A Randomized Controlled Trial

**DOI:** 10.3390/nu13072266

**Published:** 2021-06-30

**Authors:** Neda Haghighat, Damoon Ashtary-Larky, Reza Bagheri, Alexei Wong, Neda Cheraghloo, Gholamreza Moradpour, Michael Nordvall, Omid Asbaghi, Nader Moeinvaziri, Masoud Amini, Zahra Sohrabi, Frédéric Dutheil

**Affiliations:** 1 Laparoscopy Research Center, School of Medicine, Shiraz University of Medical Sciences, Shiraz 71348-14336, Iran; Neda.hag@gmail.com (N.H.); nedahaghighat@sums.ac.ir (G.M.); nmv1986@yahoo.com (N.M.); 2 Nutrition and Metabolic Diseases Research Center, Ahvaz Jundishapur University of Medical Sciences, Ahvaz 61357-15794, Iran; damoon_ashtary@yahoo.com; 3 Department of Exercise Physiology, University of Isfahan, Isfahan 81746-73441, Iran; will.fivb@yahoo.com; 4 Department of Health and Human Performance, Marymount University, Arlington, VA 22207, USA; mnordval@marymount.edu; 5 Department of Epidemiology and Biostatistics, School of Public Health, Tehran University of Medical Sciences, Tehran 1417613151, Iran; neda_cheraghloo721@yahoo.com; 6 Cancer Research Center, Shahid Beheshti University of Medical Sciences, Tehran 1416753955, Iran; omid.asbaghi@gmail.com; 7 Nutrition Research Center, School of Nutrition and Food Sciences, Shiraz University of Medical Sciences, Shiraz 71348-14336, Iran; zahra_2043@yahoo.com; 8 Université Clermont Auvergne, CNRS, LaPSCo, Physiological and Psychosocial Stress, CHU Clermont-Ferrand, University Hospital of Clermont-Ferrand, Preventive and Occupational Medicine, WittyFit, F-63000 Clermont-Ferrand, France; fred_dutheil@yahoo.fr

**Keywords:** normal weight obesity, high-protein diet, high protein snack, body composition

## Abstract

(1) Background: The favorable effects of high protein snacks on body composition and appetite status in lean and athletic populations have been illustrated previously. However, the effects of soy-enriched high protein snacks have not been investigated in women with normal-weight obesity (NWO). Consequently, we aimed at comparing the effects of six months of soy-enriched high protein snack replacement on appetite, body composition, and dietary intake in women with NWO. (2) Methods: One hundred seven (107) women with NWO [(age: 24 ± 3 yrs, BMI: 22.7 ± 2.3 kg/m^2^, body fat percentage (BFP): 38 ± 3.2%)] who were assigned to one of two groups; high protein snack (HP, *n* = 52) containing 50 g soybean or isocaloric low-protein snack (protein: 18.2 g, carbohydrate: 15 g, fat: 10 g, energy: 210 kcal) or isocaloric low protein snack (LP, *n* = 55) containing 3.5 servings of fruit (protein: <2 g, carbohydrate: ≈50 g, fat: <1 g, energy: ≈210 kcal) as part of their daily meals (as a snack at 10 a.m.), successfully completed the study interventions. Body mass (BM), body mass index (BMI), waist circumference (WC), BFP, skeletal muscle mass, dietary intake, and appetite levels were evaluated prior to and after the six-month intervention. (3) Results: Appetite (HP = −12 mm and LP = −0.6 mm), energy intake (HP = −166.2 kcal/day and LP = 91.3 kcal), carbohydrate intake (HP = −58.4 g/day and LP = 6.4 g/day), WC (HP = −4.3 cm and LP = −0.9 cm), and BFP (HP = −3.7% and LP = −0.9%) were significantly (*p* < 0.05) reduced, while skeletal muscle mass (HP = 1.2 kg and LP = 0.3 kg) significantly increased in the HP compared to the LP group, respectively. (4) Conclusions: Six months of a soy-enriched high protein snack replacement decreased appetite and improved body composition in women with NWO. Our findings suggest that soy-enriched high protein snacks are an efficacious strategy for body composition improvement.

## 1. Introduction

Normal-weight obesity (NWO) is recognized as a specific type of obesity and characterizes individuals as having normal body mass (BM) and body mass index (BMI) with an elevated fat mass (FM) (<25 (kg/m^2^) and body fat percentage ˃ 30%) and concurrent diminished lean mass [1]. It has been proposed that low dietary protein intake may be related to the lower lean mass observed in this population [2]. NWO is a widespread public health issue that may be prevalent in up to one-third of individuals of certain ethnicities [3]. For instance, a recent Iranian large population study involving 9704 individuals consisting of 2439 normal-weight adults showed that 38.5–46.2% of Iranian adults with normal BM met NWO criteria [4]. Further, the prevalence of NWO is drastically greater in women compared to men [5] suggesting that strategies for improving this condition be directed at specific cohorts.

Adipose tissue accumulation in obesity has been attributed to several factors, including increased snacking tendencies (defined as any eating occasion outside of mealtime), which may result in weight gain due to excess caloric intake [6,7]. Indeed, it has been previously reported that snacking accounts for as much as 30% of the daily energy intake [8] with women snacking more frequently than men [9] suggesting that hormonal differences may play a role in the development of NWO [10]. Because the nutrient and caloric density of snacks vary, altered body composition may be significantly influenced by macronutrient density, such as protein. Increased protein consumption is known to improve body composition, appetite control, and energy intake management [11,12]; suggesting that high protein snacks may offer useful approaches to achieve these adaptations. For instance, Astbury et al. demonstrated that high protein snacks significantly decreased dietary energy intake in lean men [13] and improved body composition in male athletes [14,15]. However, the effects of high protein snack replacement in women with NWO are currently unknown. Evidence suggests that improvements in body composition resulting from higher protein intake may result from elevated anorexigenic and reduced orexigenic hormones, leading to declines in adiposity [16,17]. Moreover, increased lean mass via muscle protein synthesis (MPS) may be enhanced by upregulating the mechanistic target of rapamycin (mTOR) signaling pathway activity [16]. It has been estimated that consumption of two to three meals a day, containing ~25–30 g of high quality protein is optimal for the stimulation of 24-h MPS in healthy adults [18]. Although prior studies have evaluated the acute effects of high protein snack replacement on satiety levels of both healthy and overweight women [19,20], there are no published investigations on long-term soy-enriched high protein snack replacement on body composition, appetite, and dietary intake. In other populations, such as Asians, vegetarians, and vegans, soy provides the main source of dietary protein [21], yet little is known about its applicability to the population in the present study. Therefore, we aimed to assess the effects of six months of soy-enriched high protein snack replacement on body composition, appetite control, and ad libitum caloric intake in women with NWO. We hypothesized that long-term soy-enriched high protein snack replacement would promote a primary outcome of improved skeletal muscle mass (SMM) and secondary outcome of appetite suppression.

## 2. Materials and Methods

### 2.1. Study Population

This parallel design (allocation ratio: 1:1) randomized clinical trial was conducted at Shiraz University of Medical Sciences (SUMS), Shiraz, Iran. Participants were recruited from the staff of companies supported by SUMS via posters and social media advertising. Participant inclusion criteria included healthy, pre-menopausal, BMI > 18.5 and <25 kg/m^2^, body fat percentage (BFP) > 30% [1,22], and women between the ages of 20 and 40 years. Exclusion criteria included history or presence of bariatric surgery, any acute or chronic diseases, psychiatric disorders, soy allergy, alcohol consumption, smoking, medications use, engaging in any high-intensity physical activity, consumption of more than 300 mg of caffeine daily (described as caffeine users) [23], as well as being pregnant and/or lactating. Further exclusion criteria were recent BM fluctuations, dietary changes, and the use of weight loss (green tea, caffeine, etc.) or other protein supplements (whey proteins, casein, etc.) within six months of the initiation of the study. Inclusion and exclusion criteria were assessed prior to the study following face-to-face meetings with each prospective participant. All study protocols were fully explained to participants who subsequently provided written consent. The study was conducted in accordance with the principles of the Declaration of Helsinki and the Ethics Committee at SUMS approved the study protocol (IR.SUMS.REC.1398.776). The present study has been registered with the Iranian Registry of Clinical Trials {(IRCT), (IRCT20170412033393N3)}.

### 2.2. Study Protocol

Participants (N = 120) who met the inclusion criteria were randomly allocated into one of two groups; a high protein snack (HP; *n* = 60) or an isocaloric low protein snack (LP; *n* = 60) that was incorporated into their daily meals using a convenience allocation. The HP group received high protein content snacks {50 g of soybeans (protein: 18.2 g, carbohydrate: 15 g, fat: 10 g, energy: 210 kcal)} while the LP group received low protein content snacks (≈3.5 servings of fruit), as desired, from the exchange list of foods based on American Diabetes Association and American Dietetic Association guidelines [24,25] (Table 1). On a daily basis, participants in the HP group were instructed to weigh 50 g of soybeans on a digital scale whereas the LP group chose 3.5 servings of fruit based on the aforementioned guidelines. The snacks for both groups contained similar calories (≈210 kcal) [24,25] and all participants were instructed to consume their snacks daily at 10 a.m (~3 h before lunch). The exchange list of foods has been frequently utilized and validated in the Iranian population [26]. Throughout the intervention, participants consumed food and thus calories ad libitum and were instructed to maintain normal dietary habits and levels of physical activity while abstaining from supplement intake. Therefore, dietary macro-and micronutrient consumption remained a free-living condition of participants for the duration of the study [13]. Body composition testing, questionnaires for 24-h dietary recalls, physical activity, and appetite were completed at baseline and after six months of the intervention. Participants also visited the laboratory on two other occasions (at the end of months 2 and 4) to report snack compliance and physical activity levels, be reminded of consuming snack meals, as well as avoid lifestyle modification. Additional snack compliance reporting and reminders were performed once per week by phone or WhatsApp software.

### 2.3. Randomization and Blinding

After baseline assessments, randomization of participants was performed through a computer-generated random number table by an independent coordinator, not otherwise involved in the study, who created an allocation sequence that assigned participants to the HP or LP groups. A dietitian subsequently assigned participants to their respective groups (the allocation sequence was concealed by the independent coordinator until the moment of assignment), provided verbal (phone) and written (WhatsApp) instruction on their snack regimens, and facilitated one-on-one laboratory interventions but therefore, could not be blinded. Therefore, study participants were aware of their treatment group for the duration of the study based on their snack allocation (HP or LP group). However, the dietitian did not participate in nor was their communication about data collection and/or analysis with any other researcher associated with this study. Further, the researchers associated with the study herein (except for the dietitian) were blinded to the treatment allocation until the database was unlocked for subsequent data analysis. At no time during the six-month intervention period did the researchers communicate with study participants.

### 2.4. Dietary Intake and Physical Activity Assessment

24-h dietary recalls were completed by all participants on three occasions over a week time period (one weekday and two weekend days) [27] prior to and at the conclusion of the six-month intervention [28]. The validity and reliability of this methodology for documenting caloric intake have been previously described [29,30,31]. Calorie and macronutrient combinations were assessed using the Nutritionist IV for Windows software program (The Hearst Corporation, San Bruno, CA). Physical activity level was recorded at baseline and six months using the international physical activity questionnaire (IPAQ) and presented as metabolic equivalent/wk (MET-min/wk) [32]. Metabolic equivalent values of 3.3, 4.0, and 8.0 were used to classify participants into low, moderate, or vigorous-intensity categories, respectively [28]. Within and between-group comparisons were made at baseline and six months for both dietary intake and physical activity levels.

### 2.5. Body Composition Assessment

Prior to arriving at the laboratory, participants fasted for 12 h (overnight and ideally following 8 h sleep), refrained from consuming alcohol and caffeinated or other diuretic beverages for 48 h before assessments [33]. Moreover, 30 min prior to assessment, participants were asked to urinate (void) completely and avoid consuming water according to standard procedures for bioelectrical impedance analysis (BIA) [34]. Height was measured using a wall-mounted stadiometer (SECA Model 222; Seca, Germany) having an accuracy of 0.1 cm. Body composition characteristics were measured by multi-frequency BIA (Inbody 270, Biospace, Korea). In advance of the BIA measurement, the participant’s palms and soles were wiped with electrolyte tissue. Participant demographics were manually entered into the BIA display by a researcher. Participants were instructed to stand on the BIA device, placing the soles of their feet on the electrodes. To determine BFP, BMI, and SMM, participants grasped the handles of the BIA unit ensuring that the palm and fingers of each hand made direct contact with the electrodes while fully extending and abducting their arms at approximately 20° [35]. Multi-frequency BIA has been shown to be a valid and reliable method [36,37], which provides acceptable body composition estimates compared to dual x-ray absorptiometry (the gold standard) [35]. Indeed, the test-retest reliability of the BIA method was high (R = 0.95 to 0.99) and has been previously described [36]. Waist circumference (WC) measurement was obtained at the noticeable waist narrowing located approximately halfway between the costal border and the iliac crest [38]. Within and between group comparisons were made at baseline and six months for all body composition measurements.

### 2.6. Appetite Assessment

Each participant’s appetite level was estimated by visual analog scales (VAS) [39] one day following body composition assessment via in-person questionnaires. VAS was completed 5 min prior to lunch (~3 h following a morning snack of the participant’s choice with ad libitum water intake), which traditionally is the largest Iranian meal [40]. The VAS questionnaire contained three questions about appetite (“How hungry do you feel?”, “How full do you feel?”, “How much would you like to eat?”) on separate 100 mm in length scales. To express their sensations of hunger and satiety, participants drew a vertical mark across the 100 mm scale with “no appetite” at one end (0 mm) and “uncontrollable appetite” (100 mm) at the other, with low, average, high, and very high demarcations in between [41]. After participants marked the questionnaires, subjective responses were converted to quantitative values according to the location of the mark ranging from 0–100 mm [41]. The numerical scores of between 0 and 100 mm were averaged from the three questions for each participant and presented as a single score which was subsequently analyzed for within and between-group differences at baseline and six months.

### 2.7. Statistical Analyses

A priori sample size calculation was conducted using the G*Power analysis software [42]. The rationale for sample size was based on our previous research, which documented significant improvements in lean mass after 12 weeks of a high protein diet in 24 women with NWO [43]. By utilizing the equation for effect size (ES) {(mean before-mean after the high protein diet)/the pooled standard deviation}, this study revealed an ES of 0.38 {(33.5–34.8)/3.45}. In the present study and based on α = 0.05, a power (1- β) of 0.80, and an ES = 0.4 (highest approximate effect size), a total sample size of at least 88 participants (*n* = 44 per group) was needed for sufficient power to detect significant changes in the primary outcome of lean mass. To account for potential participant attrition during our six-month-long investigation, additional participants (25% above recommended sample size) were recruited, thus increasing the target sample size to 110 participants (*n* = 55 per group). Descriptive statistics were presented using mean ± standard deviation (SD). Independent t-tests were used to compare means between the HP and the LP groups for each variable, while paired t-tests were used to evaluate changes over time. An analysis of covariance (ANCOVA) was used to examine the impact of groups on variables in post-test controlling for the effects of pre-test values. All assumptions were assessed and when the homogeneity of variance was not met, the parameter was estimated with robust standard errors (based on the large sample robust estimator of the covariance matrix). Cohen’s *d* effect size (ES) was calculated as post-training mean minus pre-training mean/pooled pre-training standard deviation means [44]. All analyzes were performed by SPSS (version 26) and *p*-values < 0.05 were considered as statistically significant. Figure 2 was produced using Graphpad Prism (8.4.3).

## 3. Results

### 3.1. Study Population and Compliance

**Figure 1:**
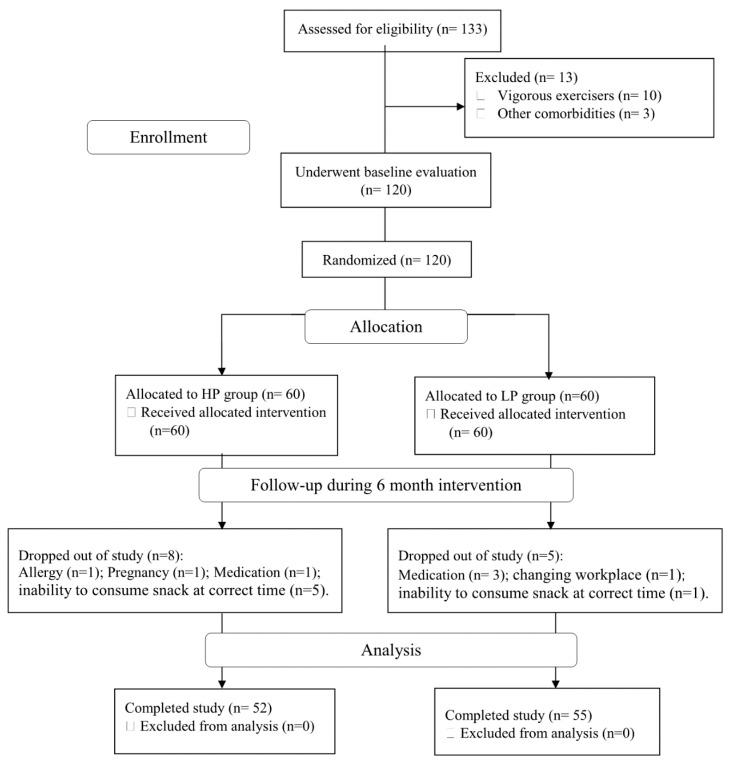
Participants flow diagram.

Between October 2018 and April 2019, we screened 133 women with NWO. After exclusion criteria were applied, 120 qualified for baseline evaluation and were subsequently randomized to either the HP (*n* = 60) or the LP (*n* = 60) groups. After randomization, eight participants in the HP group dropped out for various personal reasons. In the LP group, five participants were excluded from continuing in the study due to new medication use and an inability to consume snacks. Data are shown for the 107 participants (age: 24 ± 3 yrs and height: 166 ± 7.4 cm), consisting of 52 and 55 participants in the HP and LP groups, respectively, that successfully completed the six-month intervention (Figure 1). Participants in both groups reported no ill side effects with the dietary snacks during the duration of the study. The overall participant compliance rate was 89.1% (86.6% in the HP group and 91.6% in the LP group).

### 3.2. Body Composition

**Figure 2:**
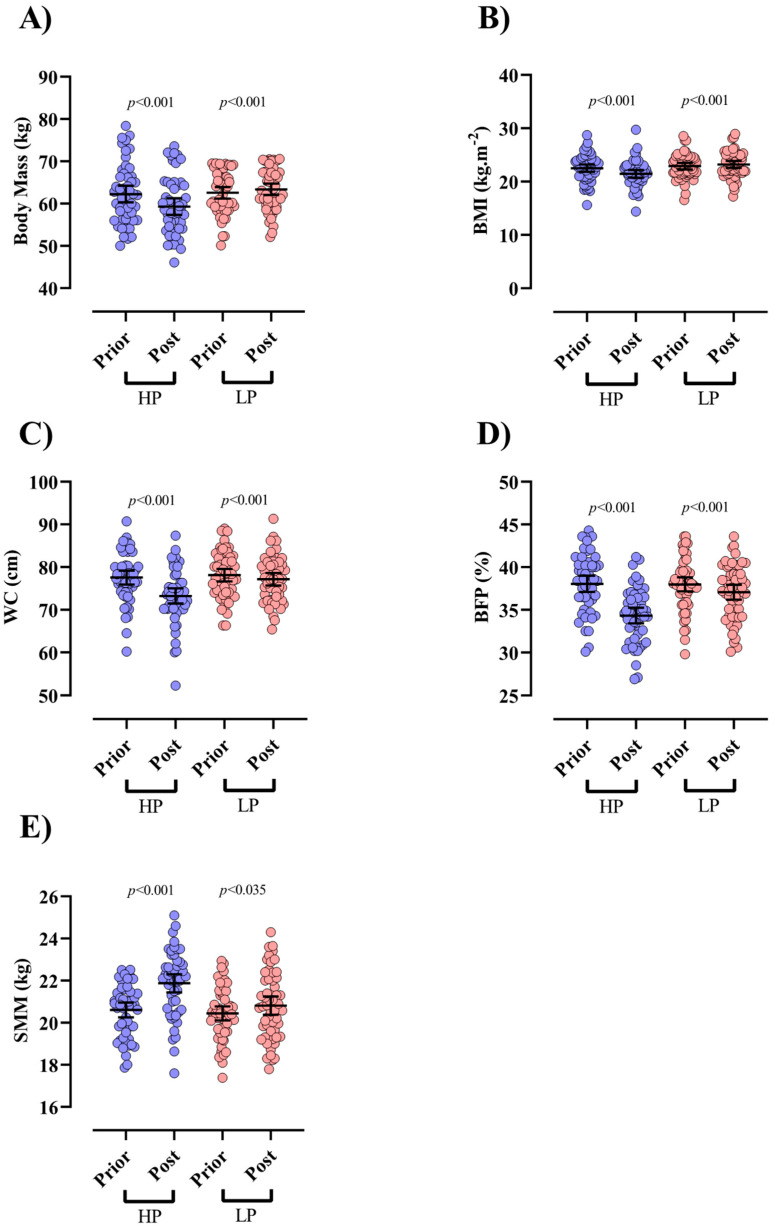
The effects of HP and LP interventions on body composition variables. (**A**) Body Mass, (**B**) BMI: body mass index, (**C**) WC: waist circumference, (**D**) BFP: body fat percentage, (**E**) SMM. Error bars indicate 95% CI. P-values of t-test are indicated above each group.

There were no significant differences in body composition variables between LP and HP groups at baseline (*p* > 0.05). Participants in the HP group experienced significant declines in BM {−2.9 kg (95% CI = −2.4 to −3.4, *p* < 0.001, d = 0.4), (Figure 2A)} and BMI {−1 kg/m^2^ (95% CI = −0.8 to −1.2, *p* < 0.001, d = 0.4), (Figure 2B)} from baseline to post-intervention. In contrast, those in the LP group experienced a significant increase in BM {0.8 kg (95% CI = 1.2 to 0.3, *p* < 0.001, d = 0.1)} and BMI [0.3 kg/m^2^ (95% CI = 0.4 to 0.1, *p* < 0.001, d = 0.1)] during the same time. Additionally, both groups significantly decreased WC {HP = −4.3 cm (95% CI = −2.8 to −5.8, *p* < 0.001, d = 0.7) and LP = [−0.9 cm (95% CI = −0.6 to −1.2, *p* < 0.001, d = 0.1), (Figure 2C)] and BFP {HP = −3.7% (95% CI = −3.3 to −4.1, *p* < 0.001, d = 1.1) and LP = −0.9% (95% CI = −0.5 to −1.2, *p* < 0.001, d = 0.3), (Figure 2D)} from baseline to post-intervention. Moreover, both groups significantly increased SMM {HP = 1.2 kg (95% CI = 1.5 to 1, *p* < 0.001, d = 0.9) and LP = 0.3 kg (95% CI = 0.7 to 0.02, *p* = 0.035, d = 0.2), (Figure 2E)} from baseline to post-intervention. ANCOVA results showed significant between-group differences for all body composition indices (*p* < 0.001, Table 2) with greater changes being noted in the HP group over time.

### 3.3. Appetite and Physical Activity

There were no significant differences in these variables between LP and HP groups at baseline (*p* > 0.05). Appetite levels in the HP group {−12 mm (95% CI = −13.2 to −10.9, *p* < 0.001, d = 2.9)} significantly decreased whereas no change was observed in the LP group pre-to post intervention. Indeed, the change in appetite in the HP was significantly greater than the LP group (*p* < 0.001, Table 2) over time. No significant differences were observed for physical activity {(pre-HP, low: 19 (36%) and moderate: 33 (63%)} vs. {post-HP, low = 21 (40%) and moderate: 31 (60%)} and {pre-LP, low: 17 (31%) and moderate: 38 (69%)} vs. {post-LP, low: 20 (36%) and moderate: 35 (63%)} from baseline to post-intervention (*p* > 0.05).

### 3.4. Dietary Intake

Results of independent t-test showed that except fiber, the difference of markers between HP and LP was not significant at baseline. Following the intervention, energy {−166.2 kcal/day (95% CI = −109 to −223.5, *p* < 0.001, d = 0.9)} and fat intake {−2.98 g/day (95% CI = −0.2 to −5.7, *p* = 0.035, d = 0.4)} significantly decreased in the HP group; while significantly increasing in the LP group {energy = 91.3 kcal/day (95% CI = 127 to 55.6, *p* < 0.001, d = 0.3) and fat intake = 4.5 g/day (95% CI = 6.6 to 2.4, *p* < 0.001, d = 0.4)}. Absolute Protein {HP = 23.5 g/day (95% CI = 26 to 21.2, *p* < 0.001, d = 3.4) and LP = 6.2 g/day (95% CI = 8.6 to 3.8, *p* < 0.001, d = 0.9)} and relative protein intake {HP = 0.4 g/kg/day (95% CI = 0.5 to 0.4, *p* < 0.001, d = 2.6) and LP = 0.09 g/kg/day (95% CI = 0.1 to 0.04, *p* < 0.001, d = 0.7)} significantly increased in both groups. Furthermore, fiber intake significantly and similarly increased over time in both groups {HP = 1.1 g/day (95% CI = 1.7 to 0.4, *p* = 0.001, d = 0.4) and LP = 1 g/day (95% CI = 1.8 to 0.1, *p* = 0.021, d = 0.2)}; while carbohydrate intake {-58.4 g/day (95% CI = −45.8 to −70.9, *p* < 0.001, d = 1.2)} significantly decreased only in the HP group {(no change in the LP group); (all nutrients are shown in Table 3)}. ANCOVA results showed significant between-group differences with absolute protein (β = 19.46, *p* < 0.001), and relative protein (β = 0.38, *p* < 0.001) were significantly higher; while fat (β = −6.31, *p* < 0.001), carbohydrate (β = −62.43, *p* < 0.001) and energy (β = −238.37, *p* < 0.001) significantly lower in HP compared to LP group (Table 2).

## 4. Discussion

We examined the effects of a six-month soy-enriched high protein snack meal containing 50 g of soybean on appetite, body composition, and dietary intake in women with NWO. Accordingly, this intervention improved body composition by increasing SMM and reducing BM, BFP, and appetite levels in this population. Although it has been previously shown that a high protein macronutrient diet favorably impacts body composition and appetite [45,46,47], the present study demonstrated that long-term soy-enriched high protein snack consumption, in the absence of other dietary modifications, offers similar benefits.

Enhancing body fat loss while maintaining SMM is a primary outcome of obesity-related interventions and therefore, the amount of SMM loss relative to reduced BM presents a biomarker of clinical efficiency [48]. Increasing the proportion of dietary protein is critical in achieving the goal of minimizing SMM loss during BM reduction strategies [46]. To that effect, a meta-analysis of 87 short-term dietary studies found a critical protein intake level exists of greater than 1.05 g/kg/day of actual BM, which was associated with 0.6 kg additional lean mass gains compared to lesser or no lean mass gain with protein intake below that level [49]. Moreover, the authors reported that in studies conducted for >12 weeks, lean mass gains increased to 1.21 kg. In the present study, BM loss observed in the HP group occurred concurrently with increased lean mass. These results are consistent with prior work by our group where an HP diet increased lean mass in women with NWO [43]. With respect to the effects of high protein intervention studies on lean mass, a meta-analysis of 24 trials has shown that when compared to a standard protein (SP) diet, a prescribed eucaloric HP diet provides modest benefits of BM reduction and in mitigating fat-free mass (FFM) reductions [50]. With respect to high protein snack replacement, Treyzon et al. revealed that a concurrent higher protein meal replacement within an elevated protein diet resulted in similar BM reductions compared to a eucaloric lower protein meal replacement plan spanning 12 weeks [51]. Similar to our results here, they also reported a significantly greater fat loss in the high protein snack group while maintaining lean mass. The discrepancy in BM reduction results from the Treyzon et al. study and ours may be explained in several ways. First, all participants in their study received a dietary plan based on a 500-kcal deficit of estimated resting metabolic rate to achieve BM loss, whereas our participants followed an ad libidum strategy, which potentially addressed satiety levels (HP group). Second, baseline protein intake among participants in our study was relatively low and the intervention length was longer than the Treyzon et al. study (six vs. three months) suggesting body composition alterations were enhanced over time in our yet-to-be elucidated upon population. With regard to caloric intake and similar to our results, a double-blind, randomized, crossover design by Astbury et al. showed that in an ad libidum condition, total daily energy intake was significantly lower when a high protein snack (whey protein and polydextrose) was consumed compared to a low protein snack (12.6 vs. 0.6 g of protein, respectively).

A potential mechanism for the observed SMM improvements following the HP intervention may be explained by dietary protein-induced alterations in protein turnover, particularly MPS [49]. It is widely acknowledged that the mechanistic target of rapamycin complex 1 (mTORC1) signaling regulates MPS in response to anabolic stimuli such as essential amino acid availability. In the present study, we used high biological value protein containing abundant essential amino acids, which may have led to an enhanced MPS [52]. Indeed, soybean is a high-quality protein that has a Protein Digestibility Corrected Amino Acid Score (PDCAAS) and Digestible Indispensable Amino Acid Score (DIAAS) [53] that are nutritionally equivalent to meat and eggs [54]. Moreover, soybean exhibits a higher amount of protein per equivalent volume of dry mass when compared to other common protein sources [55]; making soybean a relevant protein food choice to maximize muscle hypertrophy [56,57].

Dietary plans containing high protein snack replacements have been proposed as an efficient BM management approach through improved adherence in individuals with obesity [19,20,58]. To the best of our knowledge, this is the first randomized controlled trial that investigated the effects of soy-enriched high protein snack replacement in individuals with NWO. Our results suggested that compared to a low protein snack, six months of soy-enriched high protein snack replacement significantly decreases BM, BMI, and BFP in women with NWO. Similarly, it has been proposed that a high protein diet may serve as an efficient strategy to reduce FM and BFP compared to a standard macronutrient protein diet [45,46]. Indeed, Azadbakht et al. demonstrated that a combination of a high protein and low-fat diet reduced BM and WC compared to a standard high protein diet alone in overweight and obese women [59]. Moreover, we recently indicated that a eucaloric HP diet is more effective in reducing FM and BFP compared to an SP diet in women with NWO [43]. Such desirable impacts of high protein snack replacements in the present study may in part be due to a reduction in appetite and ad libitum dietary energy intake, as evidenced by our current findings. These results appear consistent with a study by Weigle et al. who observed increased dietary protein intake produces a sustained decrease in ad libitum caloric intake and theorized that the decline in caloric intake may be mediated by increased central nervous system leptin sensitivity resulting in significant BM loss [47]. It is important to note that altering a single snack meal may be more achievable and tolerable for the population in our study when compared to more drastic dietary modifications (e.g., high protein or low-fat diets) and may be considered a viable alternative for improved body composition goals.

Additionally, it has been shown that a soy-enriched meal replacement is effective in lowering BM and FM in individuals with obesity [60]. Several studies have reported that soy protein interventions in overweight and obese individuals decrease BM, WC, and BMI [61] and that FM reductions using soy products may be due to their protein, fiber, and isoflavones content [62,63]. With implications for future human trials, animal models have illustrated that adding protein to carbohydrates during dietary intake causes increased satiety and decreased food intake as fat becomes the predominant fuel source (as measured via decreased respiratory exchange ratio), which resulted in increased BM loss [64,65]. In the current study, six months of soy-enriched high protein snack replacement approximately doubled the protein to carbohydrate ratio from 0.2 to 0.38 in the HP group, indicating that other than increased satiety and decreased food intake (negative energy balance), enhanced fat metabolism may be considered among the mechanisms for reductions in BM, BMI, and BFP.

A growing body of evidence suggests that increasing dietary protein may be an effective strategy for decreasing appetite [66]. A primary methodology of several investigations included short-term feeding strategies utilizing subjective measures of appetite or analyzing the results of a single meal as the objective [11,47,67,68,69]. For example, a study by Leidy et al. indicated that consuming a high protein soy-enriched snack for three days (26 g of protein and 6 g of fat per 27 g of carbohydrates) in the afternoon elicits greater appetite suppression compared to a high-fat snack (4 g of protein and 12 g of fat per 32 g of carbohydrates) [70]. Moreover, Weigle et al. found that by implementing a eucaloric yet higher protein ad libitum condition for two weeks (increasing dietary protein from 15% to 30%), satiety was markedly increased [47]. Potential mechanisms to consider for decreased food intake and increased satiety associated with high protein diets involve increased secretion of satiety hormones such as cholecystokinin (CCK), gastric inhibitory polypeptide (GIP), glucagon-like peptide (GLP-1), peptide YY (PYY), and reduced orexigenic hormone secretion such as ghrelin [70].

We are aware that our study has some limitations including that appetite analysis was based on a VAS containing a relatively new method not widely used in the nutrition/dietetics field [71] despite its proven validity and reliability [41,72]. In addition, the pre-VAS testing snack was not the same nor did we control energy intake prior to the snack. However, prior and similarly relevant studies did not control for these variables either [47,73,74,75]. Nevertheless, future investigations should aim for stricter control protocols prior to VAS testing. Moreover, subjective interpretations of biological mechanisms do not provide the full picture of appetite control and certainly, energy intake and other variables contribute to levels of satiety and satiation [76]. Beyond the self-perceived appetite level, there is a need to determine the strength of satiety during ad libitum intake [76]. Although the study duration (six months) is a strength of our study, the lack of mid-intervention analysis limits our conclusions. Urine sample collection and analysis, which would have been a useful tool to assess dietary adherence through 24-h urinary nitrogen [77] were not performed in the present study. Furthermore, aside from the inherent differences in protein content of the high protein and ‘control’ snacks, there were also differences in carbohydrate and fat content. Therefore, at least part of the significance of our results may be due to such differences in the snack macronutrient ratios. Additionally, the protein intake of our participants at baseline was relatively low (less than 1 g protein per kg of BM) [78] and thus, our results may not be generalizable to populations with normal protein intake. We performed a per-protocol analysis, which may have biased results due to the exclusion of participants that dropped out during the follow-up intervention period. Although this type of analysis better reflects intervention effects when performed optimally, the clinical applicability of this per-protocol approach is limited if participant compliance differs substantially from one cohort to another [79]. Lastly, multi-frequency bioelectrical impedance was used to measure body composition. This method is not as precise as dual-energy x-ray absorptiometry (the gold standard) and the clinical significance of small differences has not yet been determined [80]; however, previous studies have shown that it is a valid and reliable method [35,36,37].

## 5. Conclusions

In summary, long-term consumption of a soy-enriched high protein snack replacement significantly decreased appetite, ad libitum energy intake, and improved body composition by decreasing BFP, and increasing SMM in women with NWO. Our findings underline clinical applicability that consuming a soy-enriched high protein snack replacement may provide a practical approach in controlling caloric intake and improving body composition in cohorts with NWO.

## Figures and Tables

**Table 1:** Characteristics of the soy enriched high and low protein snack meal. Abbreviations: HP, high protein; LP, low protein; g, gram.

	HP Snack (50 g Soybeans)	LP Snack (3.5 Servings Fruit)
Energy (g)	210	≈210
Protein (g)	18.2	<2
Carbohydrate (g)	15	≈50
Fat (g)	10	<1
Fiber (g)	4.6	4–6

**Table 2:** The impact of HP vs. LP on variables.

Variables	Contrast	β (SE)	95% CI	*p*-Value
Body Mass (kg)	HP vs. LP	−3.7 (0.34)	−4.46 to −3.11	<0.001
BMI (kg.m^−2^)	HP vs. LP	−1.38 (0.13)	−1.63 to −1.13	<0.001
WC (cm) #	HP vs. LP	−3.46 (0.76)	−4.98 to −1.95	<0.001
BFP (%)	HP vs. LP	−2.80 (0.27)	−3.33 to −2.26	<0.001
SMM (kg)	HP vs. LP	0.91 (0.21)	0.5 to 1.3	<0.001
Appetite (mm) #	HP vs. LP	−11.44 (0.65)	−12.73 to −10.16	<0.001
Protein (g/day)	HP vs. LP	19.46 (1.23)	17.03 to 21.89	<0.001
Fat (g/day) #	HP vs. LP	−6.31 (1.67)	−9.62 to −2.99	<0.001
Fiber (g/day)	HP vs. LP	−0.59 (0.43)	−1.45 to 0.28	0.180
Carb (g/day) #	HP vs. LP	−62.43 (6.53)	−75.38 to −49.49	<0.001
Energy (kcal/day) #	HP vs. LP	−238.37 (30.93)	−299.70 to −177.03	<0.001
Relative protein (g/kg/day)	HP vs. LP	0.38 (0.03)	0.32 to 0.44	<0.001

Abbreviations. BMI, body mass index; WC, waist circumference; BFP, body fat percentage; SMM, skeletal muscle mass (kg); HP, high protein; LP, low protein; # Parameter estimates with robust standard errors based on the original asymptotic or large sample robust, empirical, or “sandwich” estimator of the covariance matrix of the parameter estimates.

**Table 3:** Energy and macronutrients (mean ± SD).

Nutrient	Group	Pre	Post	Post-Pre	*p*-Value of Paired *t*-Test
Protein (g/day)	HP	51.37 ± 7.36	74.94 ± 6.40 *	23.58 ± 8.35 *	<0.001
	LP	48.80 ± 7.21	55.02 ± 6.30	6.22 ± 8.84	<0.001
Fat (g/day)	HP	48.87 ± 5.42	45.88 ± 8.37 *	−2.98 ± 9.94 *	0.035
	LP	46.36 ± 7.97	50.91 ± 9.56	4.55 ± 7.87	<0.001
Fiber (g/day)	HP	11.65 ± 2.82 *	12.77 ± 2.29 *	1.12 ± 2.30	0.001
	LP	13.02 ± 3.70	14.02 ± 3.03	1.00 ± 3.12	0.021
Carbohydrate (g/day)	HP	253.48 ± 39.24	195.04 ± 33.47 *	−58.44 ± 44.96 *	<0.001
	LP	247.05 ± 57.55	253.45 ± 55.55	6.40 ± 32.29	0.147
Energy (kcal/day)	HP	1659.17 ± 172.29	1492.88 ± 175.82 *	−166.29 ± 205.64 *	<0.001
	LP	1600.69 ± 239.62	1692.07 ± 239.19	91.38 ± 132.04	<0.001
Relative protein (g/kg/day)	HP	0.84 ± 0.15	1.28 ± 0.20 *	0.45 ± 0.16 *	<0.001
	LP	0.79 ± 0.14	0.87 ± 0.12	0.09 ± 0.14	<0.001

**Abbreviations.** HP, high protein; LP, low protein; * *p* < 0.05 for independent t-test, significantly difference than LP.
